# Is a CSR Policy an Equally Effective Vaccine Against Workplace Mobbing and Psychosocial Stressors?

**DOI:** 10.3390/ijerph17197292

**Published:** 2020-10-06

**Authors:** Włodzimierz Sroka, Jolita Vveinhardt

**Affiliations:** Management Department, Faculty of Applied Sciences, WSB University, Cieplaka 1c, 41-300 Dąbrowa Górnicza, Poland; jvveinhardt@wsb.edu.pl

**Keywords:** Corporate Social Responsibility, workplace mobbing, psychosocial stressors, Lithuania, Poland

## Abstract

In this study, the problem question was raised whether corporate social responsibility (CSR) is/can be an effective tool against workplace mobbing and psychosocial stressors in organizations. Therefore, the purpose of the study is to determine the prevalence of workplace mobbing in Lithuanian and Polish organizations in order to compare in which organizations the manifestation of the phenomenon is the strongest and analyzing psychosocial stressors in parallel. To achieve the purpose, 823 employees of three types of organizations were surveyed. The respondents belonged to organizations that implement the principles of corporate social responsibility, organizations that intend to become socially responsible and organizations that do not implement corporate social responsibility and do not seek to become socially responsible. The empirical study was conducted using the questionnaire “Mobbing as a Psychosocial Stressor in the Organizations Accessing and Implementing Corporate Social Responsibility—MOB-CSR”. This questionnaire is valid and reliable; the correlation relationships between subscales show interconnectedness and statistically reliable relationships. The research results were calculated using the chi-squared test and the linear regression model. Statistically reliable relationships were found between the prevalence of workplace mobbing, psychosocial work stressors and corporate social responsibility. The results of the study show that along with the weakening of variables of corporate social responsibility, the probability of workplace mobbing is increasing but CSR in itself does not ensure the prevention of workplace mobbing in the case of Lithuanian and Polish organizations. If the findings of the study are considered by the managers of organizations, this can affect both employees’ quality of life towards improvement and more transparent/purposeful implementation of corporate social responsibility, i.e., responding to the true meaning of CSR.

## 1. Introduction

Although the number of studies on corporate social responsibility (CSR) has grown in recent decades and companies are introducing socially responsible practices, employees’ safety resulting from stress caused by flawed interrelationships still remains little investigated and research findings are contradictory. For example, Svergun and Fairlie [1] state that the perception of CSR is related to less stress and greater job satisfaction of employees but such an important circumstance as job uncertainty reduces the impact of CSR on psychological safety [2] and employees may also experience stress due to over-involvement in the enterprise’s activities [3,4]. Furthermore, Frynas and Yamahaki [5] noted that current CSR theory is dominated by theories related to the external drivers of CSR and the theory is less developed in view of the organization’s internal dynamics. In other words, the focus is on how CSR policy is related to external stakeholders [6,7]. It was also observed that in response to external pressures, enterprises do not avoid manipulations focusing on image improvement in the public context [8,9] leaving internal processes in the background and paying much less attention to them [10]. This problem is especially relevant in Central and Eastern European countries where the declarative aspect overshadows the real responsibility for stakeholders [11,12].

Implementation of CSR means considerable organizational change, which is usually accompanied by certain indefiniteness, negative responses and stress [13,14]. Experienced uncertainty is a major source of psychological tension during change taking place in the organization and can become a significant condition causing friction between employees and increasing the level of antisocial interpersonal conflicts [15,16]. One of such factors causing extreme stress in the workplace is the unethical behavior of co-workers, manifesting itself as workplace mobbing [17,18]. Baillien and Witte [19] directly linked it with the change and insecurity perceived by employees. Furthermore, although in scientific research there is evidence that CSR contributes to the improvement of the well-being of employees as stakeholders, a few questions still remain unanswered; for example, how a different status of corporate social responsibility is related to consequences accompanying mobbing in different countries [20]. Usually, the emergence and spread of workplace mobbing is associated with individual and organizational variables (e.g., [17,19,21]), but it is not fully clear how and what combinations of these variables affect business organizations in different countries. Therefore, the problem of this study is raised by the following questions: is CSR/can CSR be a vaccine against workplace mobbing and psychosocial stressors in organizations and how can implementation of change in social responsibility be related to deteriorated interrelationships in different countries. In turn, the purpose of this study is to identify the prevalence of workplace mobbing in Lithuanian and Polish organizations in order to compare in which organizations the manifestation of this phenomenon is the strongest and analyzing psychosocial stressors in parallel.

Our paper is structured in the following way. Section 1 presents the literature review on CSR and mobbing. This is followed by the material and methods adopted in our study. In the next section we present the research findings and analyze them in detail. Finally, we conclude, presenting the limitations of the study and future directions of the research.

## 2. Literature Review

### 2.1. Corporate Social Responsibility and Behavior at the Workplace

CSR is a broad concept, which is discussed in different aspects in the literature. Although the first mention of CSR was in 1953 in the USA, in Bowen’s book [22] entitled “Social Responsibility of the Businessman”, the concept quickly found many followers in Europe. It is visible in European Union initiatives until now. This is based on the fulfilment of commitments to shareholders and stakeholders [23]. Social responsibility promotes the avoidance of the imbalance emerging when commitments to some stakeholders are fulfilled at the expense of other stakeholders [24]. The core value of CSR is then to maintain the economic aspects of the organization while balancing the environmental and social issues. It means that the company should act as a socially responsible organization and create a positive impact on social, environmental and economic factors [25]. Such activities require ethical leaders. On the one hand, hiring ethical leaders may be regarded as effective strategies of ethical leadership and its maintenance in the organizations. That is, however, not enough and investing in CSR activities as well as regular communication with employees on an organization’s involvement in CSR initiatives is also necessary [26]. These CSR activities are divided into two types. The practices that are related to the psychological and physiological well-being of the employees, their rights, equal opportunities and developmental needs are known as internal CSR [27,28,29]. In turn, external CSR is related to environmental and social practices that help to strengthen the firm’s legitimacy and reputation among its external stakeholders, e.g., volunteerism, cause-related marketing, corporate philanthropy and environmental and wildlife protection as well as the local community and consumers [29,30]. Some authors also examine various psychological personal characteristics of a company’s top management, such as narcissism, that may affect strategic actions and a company’s performance including its internal CSR and overall CSR behavior. One of them is diverse narcissism, which is characterized by excessive self-love, self-pride and demand for excessive admiration [31]. For example, research conducted by Tang et al. [32] on a sample of 1500 of U.S. listed firms suggested that narcissistic CEOs cared more about CSR but hubristic CEOs cared less. In turn, based on an analysis of 265 South Korean firms, Yook and Lee [33] indicated that CEO narcissism promoted CSR initiatives and CSR enhanced firm value in the capital mark. Similar findings were brought from a survey carried out in Indonesia by Ernawan and Daniel [31]. They showed that CEO narcissism had positive effects on the corporate social responsibility disclosure. One should also note that there are studies stating that narcissistic CEOs are more likely to place greater emphasis on externally oriented CSR activities such as philanthropy and environmental and wildlife protection than on internally oriented CSR activities [34]. There are also researchers (e.g., Galvin et al. [35]) who claim that narcissistic leaders, being more charismatic than the others, can be a source of inspiration to the employees to achieve their goals. As a result, a large number of managers and employees are convinced that they should follow the CSR movement but are not fully convinced that CSR activities will help a company’s performance. However, research also shows that socially responsible actions may strengthen the identification of employees with the organization [36]. This is possible, inter alia, by hiring skilled employees and a reduction of risk [37,38]. One stresses also that CSR can help managers to build more sustainable and value-creating strategies [39]. However, organizations should communicate the examples of their socially responsible actions to the employees [29]. Employees exposed to internal CSR are more engaged than those exposed only to external CSR practices [40]. However, a problem may appear when there is no balance between internal and external CSR and employees are touched by such behavior. Scheidler et al. [41] claim that inconsistent CSR strategies with larger external than internal efforts increase employees’ turnover intentions. The reason is that people treat it as a hypocrisy of the management. One should remember that people spend a lot of time at their work, which is important not only for the person’s well-being but also for the economic development of the society. On the one hand, work provides numerous economic benefits. On the other hand, however, people face various hazards at work due to chemicals, biological and physical agents, unfavorable ergonomics, allergens, a complex web of safety hazards and numerous and varied psychosocial factors [42]. In other words, the experience of working is not always pleasant and a number of cases may be observed where interpersonal relationships turn into flawed practices of mobbing [43]. It can be stated that conflicts are practically inevitable in any organization but the management has the task to both promptly resolve them and prevent them from arising to avert psychological abuse [44] as satisfactory working conditions are of a key importance for employees’ psychological well-being. The reason is that they can affect to a large extent the work performance, relationships between the employees (one should remember that dysfunction of relationships between employees has a significant impact on the quality of communication between them) and the overall quality of life [45]. It may create conditions for the occurrence of mobbing or psychological bullying [46]. As confirmed by many researchers, an unhealthy organizational culture and a dysfunctional psychological climate create favorable conditions for the occurrence of mobbing [47,48,49,50].

One of the symptoms of possible mobbing occurrence is the activities taken by the companies. Without CSR activities, both internal and external ones, a business may lose potential staff, value to employees and attractiveness to the society. It has a positive impact on job satisfaction, organizational commitment and organizational loyalty [51]. This phenomenon conclusively demonstrates the causal relationship between CSR and business. It is obvious that organizations acting in a socially responsible manner will be less prone to mobbing and vice versa. Those in which there are no rules of CSR have the higher chance for mobbing to occur. It relates also to the other aspects of a company’s activities. Furthermore, Shallcross et al. [52] state that in the case of mobbing, the behavior is usually hidden in informal networks, friendships and “loyalty” and it enables the formation of powerful mechanisms of emotional abuse.

Based on the above deliberations, we formulated the following hypotheses:

**H1**. *There will be fewer employees who will have experienced workplace mobbing in those organizations that have declared CSR unlike in organizations seeking and not seeking CSR status*.

**H2**. *There will be fewer employees who will have experienced workplace mobbing in those organizations that implement the CSR concept than in the organizations that do not intend to become socially responsible*.

### 2.2. Workplace Mobbing

One has to state that a lot of research on mobbing has been conducted so far. All of these surveys analyzed the phenomenon in different aspects, concentrating on definitions, reasons, group of risks (i.e., the employees exposed to mobbing occurrence) and first of all, the consequences of this phenomenon. In general, mobbing distinguishes itself by hostile behavior recurring for a long time, which attempts to violate the person’s psychological and physical integrity [17]. This includes a wide range of harassment actions creating an unhealthy work environment. People who experienced mobbing felt a number of negative consequences including burnout and job dissatisfaction [53,54], willingness to quit the firm [55,56] and physical problems such as insomnia and chronic fatigue [57]. In addition, this phenomenon increases organizational cynicism in the organization [58], which can hinder the organization from reaching its goals [59] as cynical employees are less loyal. It is particularly important that the solution of such problems would help prevent possible mobbing at work. Due to this fact it carries negative consequences to the victim at a psychological, social and physical level, e.g., reducing work productivity and job preoccupation [60]. One also indicates the labor environment and the performance of the institutions, which can also be affected [17]. Given these facts, the task raised for organizations is to ensure that interpersonal relationships are based on respect and become a source of satisfaction rather than stress.

We then formulated the following hypothesis:

**H3**. *Workplace mobbing and psychosocial stressors at the subscale level will be more different than similar*.

### 2.3. Corporate Social Responsibility, Workplace Mobbing and Psychosocial Stressors

According to Frynas and Yamahaki [5], CSR explains the resource-based view (RBV), agency and ethical theories, which are suitable for analyzing internal dynamics to enable the understanding of both corporate governance and social values in organizations. The causes and consequences of stress experienced by employees are explained by the work design theory [61]. In this case, two approaches are distinguished. The first, the relational perspectives approach, focuses on how work, roles and tasks are socially integrated taking into account interdependencies and interactions. The second, proactive perspective, reflects the growing importance of employees taking the initiative to foresee and create change in work performance considering the increased uncertainty and dynamism. It does not change the thesis that although there are opponents of CSR (e.g., [62]), social responsibility can limit opportunities for the manifestation of mobbing, be of service to employees’ well-being [63] and is linked with a better climate in the organization [64]. It can be added that the likelihood of avoiding mobbing increases depending on how much attention organizations pay to trust and the ethics of their activities [45]. On the other hand, CSR standards depend on the context of the country [65]; it also may be referred to as the phenomenon of mobbing. Therefore, the questions arise: how much is the phenomenon of mobbing common in different countries, is it associated with the level of development and is it connected with culture and/or religion. There is no doubt that in some countries the cases of mobbing are more common. On the other hand, there are also countries where the occurrence of mobbing is rarer. However, one should add that in the case of the latter it may be the result of so called ‘hidden mobbing’, i.e., the phenomenon really exists in the organizations but it is difficult to observe (and thus control) [66]. Cultural differences (or similarities) of countries should be regarded as significant. For example, Baguena et al. [67] claim that the cases of mobbing at the workplace in different countries substantially differ. They explain further that one of the main reasons is the different methods of assessment. In other words, some behaviors would be treated as the manifestations of mobbing in some countries while in the others they would be considered as the ones being in accordance with the widely accepted standard.

The research of Ulas et al. [68] amongst members of the Medical Faculty of Dokuz Eylul University showed that 71% of the respondents were touched by mobbing behavior. In addition, nurses had experienced such behavior more often than doctors. These findings were in line with other research conducted at the Medical Faculty of Gazi University [69], which showed the prevalence of physical violence, verbal violence and mobbing. As a result, the majority of victims of mobbing wanted to change their work. Only less than 25% of the victims reported no incidents of mobbing. Tatar and Yuksel‘s research [70], which analyzed patients at the Istanbul Faculty of Medicine, Istanbul University, brought similar results. According to them, mobbing was identified in 130 out of 300 patients who claimed to have been subjected to trauma at the workplace (43.3%). The cases presented showed the phenomenon of mobbing in one selected country only (an emerging economy), so let us analyze other, more developed countries too. Analysis of the literature shows that developed countries face the same problems with mobbing (Table 1).

Those results confirm that joint history and heritage as well as sectors may also be regarded as the important factors when analyzing the likelihood of the occurrence of mobbing in different countries. It means that the similarity of the findings on mobbing may be expected in the countries linked together by the same (or similar) historical heritage; for example, Poland and the Czech Republic or Spain and Portugal. In case of the same countries, it was observed to a higher extent (see [73]). Although Poland and Lithuania are neighboring countries with close historical and cultural links, there are a number of differences in social, economic policy and corporate governance, which can affect the manifestation of CSR. However, it is likely that the variables affecting the emergence of workplace mobbing may coincide.

Some authors have also analyzed the role of mobbing in the relationship between psychosocial stressors and their consequences. It was stated, inter alia, that mobbing actions have played a mediator role between (a) job demands and the symptoms of post-traumatic stress disorder [79], (b) tension at work and the symptoms of depression and sleep disturbances [80], (c) organizational climate and psychological health [81] and (d) social support and physical symptoms [82].

Based on those, we formulated the following hypotheses:

**H4**. *The attitude to individual aspects of CSR in Poland and Lithuania will be more different than similar*.

**H5**. *The variables affecting workplace mobbing experiences in Poland and Lithuania will coincide*.

**H6**. *CSR variables affecting the decrease of workplace mobbing in the two countries will differ*.

## 3. Materials and Methods

### 3.1. Sample

Conducting the study, a questionnaire survey was organized, which involved 823 employees of Lithuanian (*n* = 410) and Polish (*n* = 413) organizations. To achieve the purpose of the study, employees of three types of Lithuanian and Polish organizations participated in the survey; i.e., organizations that implemented the principles of corporate social responsibility, organizations that intended to become socially responsible and organizations that did not implement corporate social responsibility (CSR) and did not seek to become CSR. Characteristics of the research sample are presented in Table 2. Its analysis allows us to state that the representativeness of the sample was maintained.

### 3.2. Procedures

Having randomly selected organizations in Lithuania and Poland, the heads of the organizations were contacted regarding the possibility to conduct a survey in their organizations. After receiving verbal permissions of the managers who agreed to cooperate, the implementation of an employees’ questionnaire survey was initiated providing information in the electronic version of the questionnaire, which included the purpose of the study, employed scales and their authors, the use of collected data and publication of the results. The preamble to the questionnaire emphasized that researchers were committed to adhering to the principles of scientific ethics and highlighting the voluntary participation of a prospective respondent in the study, which could be terminated at any time. Therefore, after answering all questions of the questionnaire, it was noted that the research participant expressed no objection to participating in the survey. Research participants were guaranteed their privacy, confidentiality and anonymity.

### 3.3. Measures

The empirical study was conducted employing the questionnaire “Mobbing as a Psychosocial Stressor in the Organizations Accessing and Implementing Corporate Social Responsibility—MOB-CSR”. This instrument is valid and reliable [83]; correlation relationships between subscales show interconnectedness and statistically reliable relationships except for work content vs. environmental responsibility. The correlation relationships between the scales of the instrument, presented in Table 3, show that despite the strength of Spearman’s correlation coefficient (i.e., strong, moderate strength, weak or very weak), statistically significant relationships were found between all scales (*p* = 0.000).

To achieve the purpose of the study, employees of three types of Lithuanian and Polish organizations participated in the survey; i.e., of organizations that implemented the principles of corporate social responsibility, of organizations that intended to become socially responsible and of organizations that did not implement CSR and did not seek to become CSR. Workplace mobbing was measured in two ways: (I) when research participants identified themselves as victims of workplace mobbing by giving a positive answer to the question “Do you experience mobbing in the workplace?” and in case of the answer “yes”, being given two additional clarifying questions (How long did/does mobbing directed at you last? How often do mobbing actions directed at you repeat?) and (II) when research participants did not admit to having experienced mobbing but this could be identified based on respondents’ answers in the following subscales: communication, isolation, reputation, demography, views, damage, emotional state and intentions. Psychosocial stressors were measured by the following subscales: nature of tasks, work content, assessment, organization, management and working environment and conditions.

## 4. Results and Discussion

Hypotheses were tested by performing the chi-squared test (Table 4, Table 5 and Table 6) and using the linear regression model (Table 7). Although in general, the focus of enterprises on CSR was related to less experience of negative relationships compared with the organizations that did not seek CSR, the testing results were ambiguous (Table 4). It should be noted that the manifestation of such experiences in the group of enterprises that were just intending to become socially responsible was significantly less than in those enterprises that had already declared CSR. Even from the perspective of two countries, the trends remained similar. Thus, hypothesis H1 was rejected.

As it has been already mentioned, from the perspective of mobbing experiences, similar trends were observed in the organizations with a different status in both countries. Based on the results, the experiences of the employees of organizations that had declared CSR and those that were seeking to become CSR differed but the general trend showed that the very idea of CSR contributed to a lower risk of workplace mobbing although it did not eliminate it fully. Although the established differences between enterprises were statistically reliable, their significance differed. That is, smaller differences between all three groups of enterprises were established in Lithuania compared with Poland. In spite of this, hypothesis H2 was confirmed. One can then state that our findings confirmed the results of Wilk [84] who stated that the adopting of the CSR concept by the company reduced the occurrence of this mobbing.

Table 5 reveals the workplace mobbing and psychosocial stressor experiences. Employee experiences in Polish and Lithuanian organizations differed statistically significantly only in seven subscales from fifteen. It is significant that there were no essential differences between the countries with regard to the behavior of managers themselves and the aspects disclosing the work environment and the quality of manager-employee interaction (more precisely, the differences were not significant). However, the organization of work, the nature of tasks, assessment and the employees’ emotional state distinguished themselves considerably: the situation was more favorable for employees in Lithuanian organizations where a smaller trend of intentions to change the job was identified. Lithuanian organizations were also more tolerant, responding to employee differences related to attitudes and demographic peculiarities. Nevertheless, although experiences of workplace mobbing and psychosocial stressors at the subscale level were in part different, there were more common similarities; therefore, hypothesis H3 was rejected.

Hypothesis H4 was based on testing the attitude to individual dimensions of CSR and was confirmed. The results of the testing show that the employees of Lithuanian organizations were more positive about the initiatives related to services and their quality, customer information and safety as well as in their relationships with internal and external stakeholders. In all these cases, statistically reliable, although not equally significant, differences were identified. These findings were in line with the research of Kliestikova and Janoskova [85] who, when analyzing the consumers’ profiles in different countries, found out that Slovakia and the Czech Republic were placed in different clusters despite their common socio-cultural and historical past.

Dependent variables distinguished in the regression model are workplace mobbing in Lithuanian organizations and along with it workplace mobbing in Polish organizations (Table 7).

### 4.1. Psychosocial Stressors in the Workplace

To determine the relationships between the subscales of psychosocial stressors in the workplace (independent variable A) (i.e., factors related to the nature of tasks, work content and assessment, work organization and management, physical work environment and conditions and subscales of workplace mobbing in Lithuanian and Polish) the linear regression model was applied.

Regression equations:

MOB-LT-A = 0.794 + 0.305 × WTA + 0.283 × WAS + 0.064 × WOR + 0.092 × WCN.

MOB-PL-A = 0.370 + 0.185 × WTA + 0.079 × WCT + 0.160 × WAS + 0.233 × WMA + 0.130 × WEN.

Strengthening of such negative factors as the negative nature of tasks, incorrect work assessment, inappropriate work organization and poor working conditions in Lithuanian organizations was accompanied by the increase of the probability of workplace mobbing in Lithuania. Workplace mobbing was most affected by variables such as the nature of tasks and work assessment while work management and working environment in principle did not have a significant influence.

Along with the worsening of inadequate nature tasks, work content that did not correspond to the job description, biased work assessment, incompetent work management, a work environment that did not meet the psychological and physical criteria in Polish organizations, the probability of workplace mobbing in Poland also increased. In this case, the greatest positive effect could be expected by rehabilitating work management, the nature of tasks and work assessment. Although some variables (except for work organization and working conditions) coincided in Lithuania’s and Poland’s case, their significance differed. Thus, this indicated that H5 must be rejected.

### 4.2. Corporate Social Responsibility

In order to identify the relationships between the subscales of corporate social responsibility (independent variable B), the linear regression analysis was performed.

Regression equations:

MOB-LT-B = 3.446 − 0.091 × RSQ − 0.098 × RCH − 0.125 × RRS − 0.279 × RRE − 0.136 × ERS.

MOB-PL-B = 4.416 − 0.184 × RCH − 0.113 × RRS − 0.151 × RRE − 0.201 × ERS.

In the case of Lithuanian organizations, it was established that as services and their quality, customer information, health and safety, responsibility in relationships with the society and responsibility in relationships with employees as well as employees’ responsibility towards customers separately one by one were worsening, the probability of the manifestation of workplace mobbing in Lithuania was increasing. However, the influence of the variables differed; responsibility in relationships with employees, employees’ responsibility towards customers and responsibility in relationships with the society had the greatest influence. That is, greater social responsibility in these areas also influenced the reduction of employees’ negative experiences.

In Polish organizations, as customer information, health and safety, responsibility in relationships with the society and responsibility in relationships with employees as well as employees’ responsibility towards customers separately one by one were worsening, the prevalence of mobbing in employee interrelations might increase. In the case of Polish organizations, employees’ responsibility towards customers, customer information, health and safety and responsibility in relationships with employees had the greatest impact. The comparison of the two countries revealed differences in the strength of the impact of the relationships with both internal and external stakeholders while services and their quality were not significant in Polish organizations at all; therefore, hypothesis H6 was rejected.

The results of this study confirmed that, in principal, CSR serves as an ideology and a practical tool contributing to increasing employees’ safety and psychological well-being. However, the similar trends recorded in the two different countries suggest that CSR alone is not an unequivocally effective vaccine to protect organizations from workplace mobbing because, in practice, approaches to factors influencing this phenomenon differ.

Research results (H3) demonstrated the existence of a similar approach of organizations to environmental protection initiatives and relationships with customers and clients of enterprises in both countries. It showed orientation to the economic aspects of CSR and demonstrated concern about environmental protection issues relevant to the society but at the same time also revealed significant differences in the relationship with internal and external stakeholders.

It is interesting that, in the case of Lithuania for example, a positive impact of increasing social responsibility in such areas as relationships with employees, relationships with the society and employees’ responsibility towards customers on reducing workplace mobbing was established. This can be explained as an indirect effect of strengthening the general CSR policy.

## 5. Conclusions

This study grounds a critical approach to CSR practice, protecting employees from workplace mobbing as a psychological stressor. To better understand why the CSR ideology that has gained popularity does not always help to protect employees from mobbing and psychological stress experienced during it, several factors related to the enterprise’s internal dynamics and national differences of CSR need to be considered. On the one hand, the implementation of CSR initiatives can reduce the risk of workplace mobbing but the national context must be taken into account. That is, knowledge of the state of CSR in a particular country can save a lot of time and money and ensure a more precise rehabilitation of the organization’s state. That is, *inter alia*, because there is no doubt that the importance of being socially responsible in business has been substantially increased recently. At the same time, mobbing is a serious problem of modern societies. It affects both the employees of the companies and their social environment as well as the work process. First of all, it has very negative consequences for the employees who face this phenomenon. Those consequences may be really very serious and can be manifested, *inter alia*, by reducing work productivity, burnout and job preoccupation. Solutions therefore need to be found. Given this fact, our study points out the extensiveness of the problem in modern organizations operating in two neighboring countries, i.e., Poland and Lithuania, trying to answer whether a CSR policy is an equally effective vaccine against workplace mobbing. These findings give some suggestions on how to behave in case of mobbing.

There are several contributions to the theory of our study. First of all, this paper represents a contribution to understanding the status of CSR and the need to enlighten the impact that socially responsible practices can have on the prevention of workplace mobbing, i.e., whether they can be an effective vaccine against this phenomenon. In addition, it presents the findings of unique quantitative research related to the phenomenon of workplace mobbing at Polish and Lithuanian organizations. It has then an international approach, enhancing the understanding of the workplace mobbing phenomenon in the cultural context of Central European countries. In addition, the research is based on the analysis of a large sample containing 823 respondents. Another contribution is that it increases the understanding of mobbing in the context of the CSR concept in the companies from the public and private sectors. Our research may also contribute to the development of socially responsible policies and procedures that then should be shared with all the employees. Furthermore, one cannot forget that due to the large research sample, our findings can be used for the formulation of research hypotheses by other scholars. This research could have an impact on both organizations and persons working with victims of mobbing, enabling them to better understand the causes of mobbing and ways of helping to ensure employees’ well-being and a safe work environment. It seems to us that it requires from the managers an open managerial approach, which requires, inter alia, clearly defining mobbing behaviors in the workplace. Fortunately, the research sample used in both countries (in relation to gender and age of respondents, size of the companies, seniority in the organizations, functions possessed by the respondents, etc.) is that one can state that its representativeness was maintained.

Of course, our study has some limitations. First of all, it reveals the results obtained in two neighboring countries only with a similar historical and cultural heritage. Therefore, in the future, it would be worthwhile to conduct the study by selecting more countries and to make comparative research, e.g., Western countries vs. Central European ones. Secondly, though a quantitative study generates interesting associations among the particular factors being analyzed, a qualitative study that would also explore the reasons would be very interesting, adding richness and depth to the findings of our survey. We also think that some kind of cyclical research to be done every 3–5 years would be recommended to see in which direction the situation in both countries evolves.

## Figures and Tables

**Table 1:** Research on mobbing in developed countries—literature review.

Authors	Research Sample	Aim of the Research	Type of Research	Findings
Meseguer de Pedro et al. [71]	396 Spanish workers from an agro fruit sector in the region of Murcia	Analysis of the different consequences of the phenomenon of mobbing on the health of the employees	Questionnaire survey	A strong link between mobbing and experienced psychosomatic symptoms was also found but the effect on absenteeism was not significant.
Motlova and Lemrova [72]	496 employees of the health care facilities in the Czech Republic	Analysis of mobbing in the workplace	Questionnaire survey	33% of respondents often or always perceived at least one type of mobbing, mostly gossip, humiliation and accusations. The most common reactions of victims were feelings of sadness, stress and worry. There was no difference in the frequency of hostile behavior in public and private facilities. In addition, there was no association with age or time effects in health and the health care facility.
Mulder et al. [73]	161 Dutch regional government employees	Analysis of victims’ perceived responsibility and bystanders’ anticipated risk of being victimized themselves	Questionnaire survey	“In the strong (vs. weak) responsibility condition, women reported less sympathy and more anger and men only more anger, which resulted in lower intention“ (p. 304) to help. In addition, a positive effect of the responsibility on men’s intentions to help was identified. Together, men demonstrated greater anger; while women, fear.
Cakirpaloglu et al. [74]	1757 employees from the state and the private sector in Czech Republic	Description and analysis of the psychological occurrence, modes of expression and the most common psychological effects in employment in the Czech Republic	Questionnaire survey	16.3 % of a prevalence of mobbing within selected regions of the Czech Republic. Victims “suffering from various mental health problems, especially anxiety and depression”(p. 66).
Figueiredo-Ferraz et al. [75]	372 Spanish employees working with people with intellectual disabilities at 61 job centers in the Valencian community	Analysis of the influence of mobbing on depressive symptoms in a sample of employees working with people with intellectual disabilities (ID)	Longitudinal study	Employees who experienced attacks at least once a week and that lasted at least six months had more depressive syndromes unlike those who were abused for a shorter period of time or less than once a week.
Stanisławska et al. [76]	418 Polish employees representing civil court officials (30.86%), the healthcare sector (49.76%) and supermarket chains (19.38%)		Questionnaire survey	The highest intensity of mobbing was found among supermarket employees and health care professionals (in the latter case, the higher intensity was related to longer seniority). Meanwhile, the lowest intensity of mobbing was found among court employees.
Giaccone and di Nunzi [77]	EU member states	Prevalence of mobbing in EU member states	Questionnaire survey	The EU 28 average is 14%; i.e., such a share of employees report that they have experienced mobbing at the workplace. Mobbing experiences in the Baltic, Central, Western and Nordic countries exceed the EU average (in Austria, the Czech Republic and Finland mobbing was experienced by over 20% of employees). Meanwhile, in Croatia, mobbing was experienced by 12% of employees; in Cyprus, by 6%.
da Silva and Portelada [17]	3227 nurses from health institutions in Portugal	Assessment of the existence, frequency and intensity of mobbing within the Portuguese nurse population and its impact on their well-being and interpersonal relationships	Questionnaire survey	On average, every nurse undergoes 11 aggression conducts in their main place of work. The types of aggression included communication blockage and being discredited at work. “Almost half of the victims claim to have had health problems as a result of having suffered mobbing“(p. 2797).
Goralewska-Slonska [78]	180 Polish students (both full-time and part-time)	Determination of the relationship of experiencing mobbing with psychological gender dimensions and occupational burnout	Questionnaire survey	Research has identified the link between mobbing experiences and occupational burnout. It was also “revealed that there was no connection between the experience of mobbing and the psychological gender dimension—femininity, while it was noticed that at the level of statistical tendency, there was the relationship between the experience of mobbing and the psychological gender dimension—masculinity“ (p. 168–167).

**Table 2:** Characteristics of the research sample.

	Lithuania		Poland		Total	
	Quantity	%	Quantity	%	Quantity	%
Status of the organization						
Private sector	197	48.0%	204	49.4%	401	48.7%
Public sector	213	52.0%	209	50.6%	422	51.3%
Total	410	49.8%	413	5022%	823	100%
Social responsibility of the organization						
Seeks to become socially responsible	93	22.7%	153	37.0%	246	29.9%
Is socially responsible	244	59.5%	174	42.1%	418	50.8%
Does not seek to become socially responsible	73	17.8%	86	20.9%	159	19.3%
Total	410	49.8%	413	50.2%	823	100%
Gender						
Male	154	37.6%	215	52.1%	369	44.8%
Female	256	62.4%	198	47.9%	454	55.2%
Total	410	49.8%	413	50.2%	823	100%
Age						
18–25 years	190	46.3%	55	13.3%	245	29.7%
26–30 years	62	15.1%	68	16.5%	130	15.8%
31–35 years	39	9.5%	62	15.0%	101	12.3%
36–40 years	25	6.1%	85	20.5%	110	13.4%
41–45 years	29	7.1%	78	18.9%	107	13.0%
46–50 years	27	6.6%	37	9.0%	64	7.8%
51–60 years	27	6.6%	21	5.1%	48	5.8%
Over 61 years	11	2.7%	7	1.7%	18	2.2%
Total	410	49.8%	413	50.2%	823	100%
Education						
Higher university (Bachelor: university, institute, academy)	208	50.7%	112	27.1%	320	38.9%
Higher non-university (professional Bachelor: college)	80	19.5%	55	13.3%	135	16.4%
Unfinished higher educational institution	58	14.1%	36	8.7%	94	11.4%
Upper secondary	19	4.6%	43	10.4%	62	7.5%
Vocational	18	4.4%	79	19.1%	97	11.8%
Secondary	25	6.1%	79	19.1%	104	12.6%
Primary	2	0.6%	9	2.3%	11	1.4%
Total	410	49.8%	413	50.2%	823	100%
Seniority at the organization						
Up to 1 year	58	14.1%	22	5.3%	80	9.7%
From 1 to 3 years	103	25.1%	73	17.7%	176	21.4%
From 4 to 7 years	83	20.2%	74	17.9%	157	19.1%
From 8 to 10 years	35	8.6%	61	14.8%	96	11.7%
From 11 to 15 years	37	9.0%	60	14.5%	97	11.8%
From 16 to 20 years	34	8.4%	61	14.8%	95	11.5%
From 21 years and more	60	14.6%	62	15.0%	122	14.8%
Total	410	49.8%	413	50.2%	823	100%
Status of employee						
Top level manager	15	3.7%	41	9.9%	56	6.8%
Middle level manager	57	13.9%	46	11.1%	103	12.5%
Low level manager	42	10.2%	34	8.3%	76	9.3%
Ordinary employee (does not have employees)	262	63.9%	176	42.6%	438	53.2%
Worker	34	8.3%	116	28.1%	150	18.2%
Total	410	49.8%	413	50.2%	823	100%
Job specifics						
Provision of services, I directly communicate with customers, interested persons	310	75.6%	242	58.6%	552	67.1%
I do technical, physical work	100	24.4%	171	41.4%	271	32.9%
Total	410	49.8%	413	50.2%	823	100%
Size of the organization						
Very small (up to 10 employees)	85	20.7%	87	21.1%	172	20.9%
Small (more than 10 but less than 50)	161	39.3%	133	32.2%	294	35.7%
Medium sized (from 50 to 250 employees)	100	24.4%	122	29.5%	222	27.0%
Large (over 250 employees)	64	15.6%	71	17.2%	135	16.4%
Total	410	49.8%	413	50.2%	823	100%
Marital status						
Lonely	116	28.3%	107	25.9%	223	27.1%
Married	125	30.5%	191	46.2%	316	38.4%
Divorced	35	8.5%	38	9.3%	73	8.9%
Living with a partner	134	32.7%	77	18.6%	211	25.6%
Total	410	49.8%	413	50.2%	823	100%

**Table 3:** Correlation relationships between workplace mobbing, psychosocial stressors and corporate social responsibility scales in the joint sample of organizations of both countries (*N*min = 823; *N*max = 823).

Factors	Scales	Factors Related to the Behavior of a Socially Responsible Organization	Factors Related to the Behavior of a Socially Responsible Employee
Workplace mobbing	Factors related to employee interrelationship	–0.570 ** *p* = 0.000	–0.410 ** *p* = 0.000
Psychosocial stressors in the workplace	Factors related to the nature of tasks, work content and assessment	–0.393 ** *p* = 0.000	–0.431 ** *p* = 0.000
	Factors related to work organization and management	–0.704 ** *p* = 0.000	–0.240 ** *p* = 0.000
	Factors related to physical working environment and conditions	–0.751 ** *p* = 0.000	–0.271 ** *p* = 0.000

Notes: * statistical significance level = 0.05; ** statistical significance level = 0.01. Spearman’s correlation coefficient: 0.6 < r ≤ 0.8 (strong relations), 0.4 < r ≤ 0.6 (moderate strength relations), 0.2 < r ≤ 0.4 (weak relations), 0.1 ≤ r ≤ 0.2 (very weak relations).

**Table 4:** Workplace mobbing experience in Lithuanian and Polish organizations.

Workplace Mobbing Experience		Organizations that Implement the Principles of Corporate Social Responsibility		Organizations that Intend to Become Socially Responsible		Organizations that Do not Implement Corporate Social Responsibility and Do not Seek to Become CSR		Chi-squared Test Results	
		LT, *n* = 244 PL, *n* = 174		LT, *n* = 93 PL, *n* = 153		LT, *n* = 73 PL, *n* = 86			
		Quantity	%	Quantity	%	Quantity	%	χ^2^	*p*
Lithuania, *n* = 410	Did not experience	227	93.0	88	94.6	62	84.9	6.144	0.046 *
	Experienced	17	7.0	5	5.4	11	15.1		
Poland, *n* = 413	Did not experience	159	91.4	143	93.5	64	74.4	22.072	0.0001 **
	Experienced	15	8.6	10	6.5	22	25.6		

Notes: * statistical significance level = 0.05; ** statistical significance level = 0.01. LT—Lithuania, PL—Poland.

**Table 5:** Workplace mobbing and psychosocial stressors in Lithuanian and Polish organizations: the subscale level.

Workplace Mobbing and Psychosocial Stressors	Experience	Lithuania (*n* = 410)		Poland (*n* = 413)		Chi-squared Test Results	
		Frequencies	%	Frequencies	%	χ^2^	*p*
Employee communication	Did not experience	338	82.4	346	83.8	0.262	0.608
	Experienced	72	17.6	67	16.2		
Employee isolation	Did not experience	362	88.3	356	86.2	0.811	0.368
	Experienced	48	11.7	57	13.8		
Employee reputation	Did not experience	338	82.4	328	79.4	1.216	0.270
	Experienced	72	17.6	85	20.6		
Employee demography	Did not experience	367	89.5	345	83.5	6.300	0.012 *
	Experienced	43	10.5	68	16.5		
Employee views	Did not experience	387	94.4	365	88.4	9.436	0.002 **
	Experienced	23	5.6	48	11.6		
Damage experienced by employees	Did not experience	362	85.9	355	86.0	0.002	0.966
	Experienced	58	14.1	58	14.0		
Employees’ emotional state	Did not experience	220	53.7	151	36.6	24.291	0.0001 **
	Experienced	190	46.3	262	63.4		
Employee intentions	Did not experience	278	67.8	224	54.2	15.920	0.0001 **
	Experienced	132	32.2	189	45.8		
Nature of tasks	Did not experience	219	53.4	154	37.3	21.592	0.0001 **
	Experienced	191	46.6	259	62.7		
Work content	Did not experience	57	13.9	46	11.1	1.436	0.231
	Experienced	353	86.1	367	88.9		
Work assessment	Did not experience	310	75.6	200	48.4	64.511	0.0001 **
	Experienced	100	24.4	213	51.6		
Work organization	Did not experience	271	66.1	241	58.4	5.249	0.022 *
	Experienced	139	33.9	172	41.6		
Work management	Did not experience	297	72.4	274	66.3	3.598	0.058
	Experienced	113	27.6	139	33.7		
Working environment	Did not experience	266	64.9	270	65.4	0.022	0.881
	Experienced	144	36.1	143	34.6		
Working conditions	Did not experience	197	48.0	207	50.1	0.354	0.552
	Experienced	213	52.0	206	49.9		

Note: * statistical significance level = 0.05; ** statistical significance level = 0.01.

**Table 6:** Corporate social responsibility in Lithuanian and Polish organizations: the subscale level.

Corporate Social Responsibility	Approval	Lithuania (*n* = 410)		Poland (*n* = 413)		Chi-squared Test Results	
		Frequencies	%	Frequencies	%	χ^2^	*p*
Services and their quality	Disagrees	52	12.7	85	20.6	9.250	0.002 **
	Agrees	358	87.3	328	79.4		
Customer information, health and safety	Disagrees	62	15.1	103	24.9	12.372	0.001 **
	Agrees	348	84.9	310	75.1		
Environmental responsibility	Disagrees	129	31.5	130	31.5	0.003	0.997
	Agrees	281	68.5	283	68.5		
Responsibility in relationships with the society	Disagrees	73	17.8	127	30.8	18.744	0.0001 **
	Agrees	337	82.2	286	69.2		
Responsibility in relationships with employees	Disagrees	85	20.7	120	29.1	7.622	0.006 **
	Agrees	325	79.3	293	70.9		
Employees’ responsibility towards customers	Disagrees	49	12.0	62	15.0	1.652	0.199
	Agrees	361	88.0	351	85.0		
Employees’ relationships with customers	Disagrees	60	14.6	63	15.3	0.062	0.803
	Agrees	350	85.4	350	84.7		

Note: * statistical significance level = 0.05; ** statistical significance level = 0.01.

**Table 7:** Workplace mobbing in Lithuanian and Polish organizations as a dependent variable.

		Dependent Variable (FEIR)							
A Independent variable Psychosocial stressors in the workplace		**Workplace Mobbing in Lithuania (MOB-LT)**				**Workplace Mobbing in Poland (MOB-PL)**			
		**R**	**R^2^**	**R^2^ revised**	**Reliability**	**R**	**R^2^**	**R^2^ revised**	**Reliability**
		0.825	0.681	0.676	0.000	0.737	0.543	0.535	0.000
		Non-standardized beta coefficient	Standardized beta coefficient	t	ANOVA reliability	Non-standardized beta coefficient	Standardized beta coefficient	t	ANOVA reliability
(Constant)		0.794		3.281	**0.001**	0.370		3.200	**0.001**
FNCA	WTA. Nature of tasks	0.305	0.363	10.076	**0.000**	0.185	0.260	4.375	**0.000**
	WCT. Work content	0.005	0.006	0.194	0.846	0.079	0.094	2.070	**0.039**
	WAS. Work assessment	0.283	0.440	10.838	**0.000**	0.160	0.256	4.467	**0.000**
FWOM	WOR. Work organization	0.064	0.092	1.932	**0.050**	–0.036	–0.048	–0.874	0.382
	WMA. Work management	0.014	0.021	0.427	0.670	0.233	0.288	5.202	**0.000**
FPEC	WEN. Working environment	–0.042	–0.059	–1.403	0.161	0.130	0.170	3.034	**0.003**
	WCN. Working conditions	0.092	0.134	3.293	**0.001**	0.060	0.076	1.383	0.168
B Independent variable Corporate social responsibility		**R**	**R^2^**	**R^2^ revised**	**Reliability**	**R**	**R^2^**	**R^2^ revised**	**Reliability**
		0.545	0.297	0.285	**0.000**	0.712	0.507	0.498	0.000
		Non-standardized beta coefficient	Standardized beta coefficient	t	ANOVA reliability	Non-standardized beta coefficient	Standardized beta coefficient	t	ANOVA reliability
(Constant)		3.446		18.823	**0.000**	4.416		29.988	0.000
FOSB	RSQ. Services and their quality	–0.091	–0.131	–1.936	**0.050**	–0.086	–0.105	–1.714	0.087
	RCH. Customer information, health and safety	–0.098	–0.146	–2.083	**0.038**	–0.184	–0.230	–3.525	0.000
	REN. Environmental responsibility	0.017	0.028	0.452	0.651	0.044	0.055	1.054	0.293
	RRS. Responsibility in relationships with the society	–0.125	–0.184	–2.664	**0.008**	–0.113	–0.131	–2.161	0.031
	RRE. Responsibility in relationships with employees	–0.279	–0.406	–6.130	**0.000**	–0.151	–0.192	–2.988	0.003
FESB	ERS. Employees’ responsibility towards customers	–0.136	–0.194	–4.044	**0.000**	–0.201	–0.257	–5.559	0.000
	ERL. Employees’ relationships with customers	–0.023	–0.029	–0.596	0.552	0.014	0.016	0.397	0.692

Notes: FEIR, factors related to employee interrelationship; FNCA, factors related to the nature of tasks, work content and assessment; FWOM, factors related to work organization and management; FPEC, factors related to the physical working environment and conditions; FOSB, factors related to the behavior of a socially responsible organization; FESB, factors related to the behavior of a socially responsible employee.
